# Editorial: Autoantibodies

**DOI:** 10.3389/fimmu.2019.00484

**Published:** 2019-04-02

**Authors:** Rikard Holmdahl, Falk Nimmerjahn, Ralf J. Ludwig

**Affiliations:** ^1^Section of Medical Inflammation Research, Department of Medical Biochemistry and Biophysics, Karolinska Institute, Stockholm, Sweden; ^2^Department of Biology, Institute of Genetics, University of Erlangen-Nürnberg, Erlangen, Germany; ^3^Lübeck Institute of Experimental Dermatology and Center for Research on Inflammation of the Skin, University of Lübeck, Lübeck, Germany

**Keywords:** autoantibody, pemphigus, pemphigoid, arthritis, pathogenesis, biomarker, diagnos, treatment

## Autoantibodies

**Figure 1:**
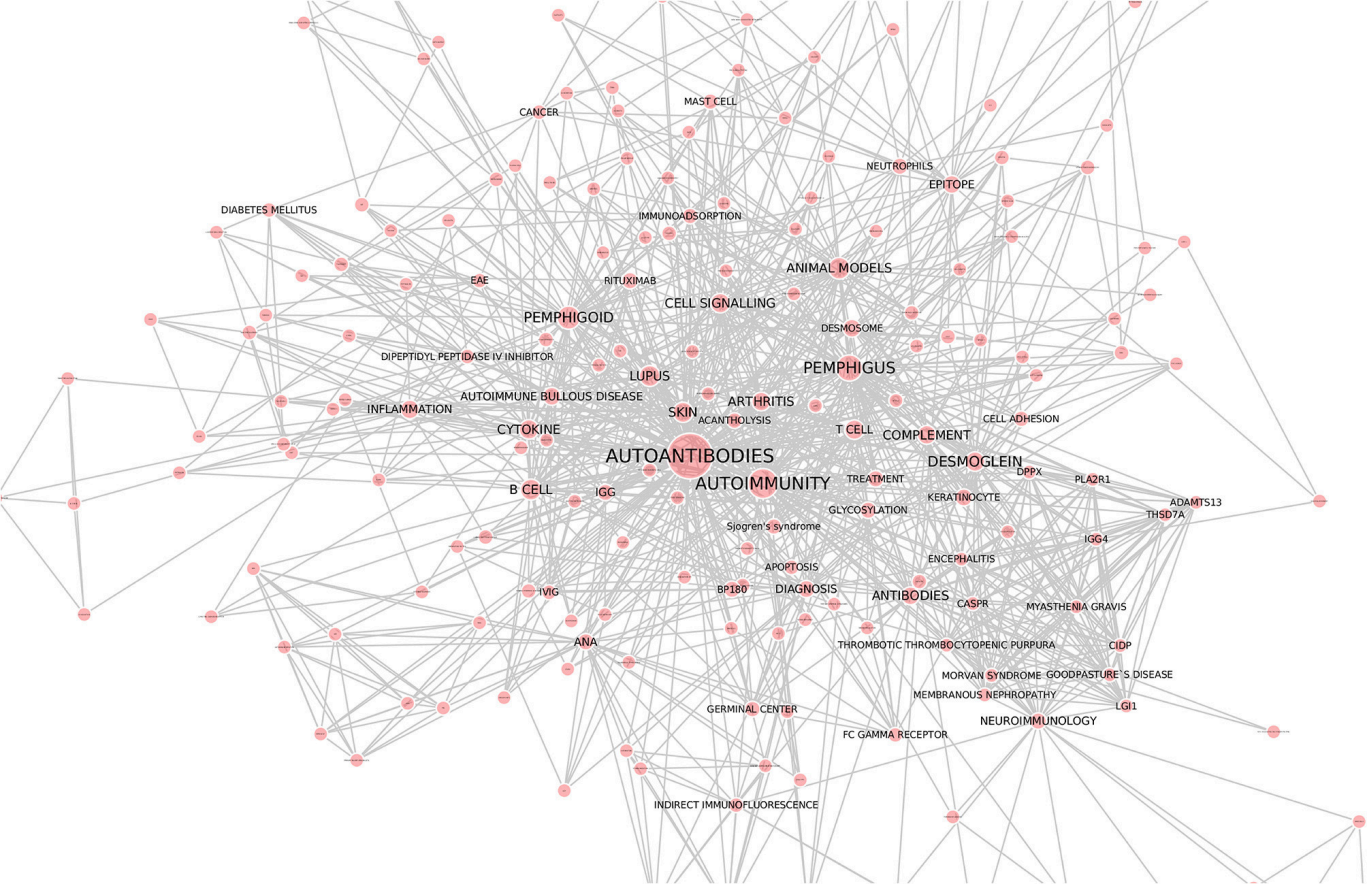
Interaction network of articles within the Research Topic “Autoantibodies”. Keywords of all 88 articles were downloaded from the website of the Research Topic “Autoantibodies” (https://www.frontiersin.org/research-topics/6220/autoantibodies). Cytoscape (https://cytoscape.org/) was used to draw the interaction map. Each line represents an interaction among the keywords, whereas the size of the red circles correlates to the number of times the respective keyword was mentioned. We thank Dr. Yask Guta (University of Lübeck) for generating this figure.

Autoantibodies have become a popular research topic with a constantly growing number of research reports. The increasing interest from the scientific community is also reflected by the high number of articles within this Research Topic, which are illustrated by an interaction map that has been drawn by using the keywords of all articles from this collection (Figure 1). These articles, selected from the theme of this topic, cluster around “Autoantibodies” and “Autoimmunity.” Clustering was also observed for specific diseases, namely, pemphigus and pemphigoid, lupus, arthritis, and neuroimmunology. Cytokines, B cells, cell signaling, and the complement cascade are the focus of many manuscripts within this Research Topic. This clustering was the basis for the selection of the manuscripts discussed in this editorial.

## Pemphigus and Pemphigoid

We received a number of submissions on the topics of pemphigus and pemphigoid, which are characterized and caused by autoantibodies to structural proteins of the skin [(1, 2); Liu et al.]. After binding to their target antigens, these autoantibodies directly (in the case of pemphigus) or indirectly (in the case of pemphigoid) cause skin blistering, which is the common clinical denominator of these diseases. Diagnosis is based on the clinical presentation, the detection of autoantibodies and/or complement deposits in the skin (detected by direct immunofluorescent (IF) microscopy), as well as the serological detection of the autoantibodies (3). For both pemphigus and pemphigoid systemic immunosuppression corticosteroids are still the main treatment. However, the lack of efficacy and/or the adverse events contribute to the medical burden of these diseases, which have an overall high unmet medical need (4). Within this Research Topic “Autoantibodies,” insights into the pathogenesis, as well as novel biomarkers and treatments, are presented with the prospect that they might improve the diagnosis and treatment of pemphigus and pemphigoid.

## Systemic Lupus Erythematosus

Systemic lupus erythematosus (SLE) is a complex and multifactorial systemic autoimmune disease that primarily affects young women. The chronic inflammatory processes triggered during this disease can affect a variety of organ systems, including the skin, blood vessels, kidneys, and joints. Loss of humoral tolerance toward nuclear antigens such as RNA, DNA, and histones is one hallmark of the disease, although the direct contribution of autoantibodies to the disease pathology in humans is still controversial. However, novel treatments targeting autoantibody-producing plasma cells have shown promising effects in patients with refractory SLE (5). In addition, novel insights into the activation and expansion of polyclonal autoreactive B cell responses during SLE in humans have emphasized the tight connection between the loss of humoral tolerance and disease activity (6). More direct evidence for the critical role of autoantibodies in SLE pathology is provided by animal model systems in the study of lupus nephritis, which have clearly demonstrated that the autoantibody-dependent activation of innate immune effector cells is a major factor for kidney and lung inflammation. With respect to the genetic factors involved in the loss of humoral tolerance to nuclear antigens, the loss or impaired signaling of the inhibitory effector FcgRIIb has been shown to lead to an increased level of autoantibody production by B cells and a decreased threshold for the activation of innate immune effector cells (7). In line with the studies in mice, a non-functional FcgRIIb variant has been shown to be a genetic risk factor for SLE development in humans (8, 9). However, it is also clear that multiple factors contribute to SLE development, including defects in apoptosis or enhanced TLR signaling (10, 11). Within the Research Topic “Autoantibodies,” Weissenburger et al. provided new insights into how mutations in the deoxyribonuclease 1-like 3 gene lead to the massive production of autoantibodies against double stranded DNA. Moreover, Biermann et al. demonstrated that autoantibodies for secondary necrotic cells allow the identification of patients with SLE. With regard to innate immune effector cells, a decreased phagocytic capacity, resulting in the prolonged presence of dying cells in the body, has also been suggested to contribute to disease development (12). In summary, many pieces of the SLE puzzle have fallen into place and suggest that the loss of humoral tolerance is not simply a side effect of SLE but is rather an active player in the pathogenesis of SLE.

## Arthritis

Rheumatoid arthritis (RA) is one of the most common autoimmune diseases and has a large socioeconomic importance. The role of autoantibodies, such as rheumatoid factors (RF), has been instrumental in the classification and the investigation on the causes and pathogenesis of the disease. More recently, additional autoantibodies, such as antibodies to citrullinated proteins (ACPA), have been described. The successful treatment with antibodies targeting B cells, reviewed by Hoffmann et al., have been key to the revival of the belief of the major role of B cells in RA and in several other autoimmune diseases. As in most autoimmune diseases, these autoantibodies appear years before the clinical onset of the disease. Sieghart et al. analyzed the isotype distribution of the different RA autoantibodies in early and established RA and showed that both the ACPA and RF of the IgG isotypes are specific for diagnosis but that the analysis of the IgM isotype increased the sensitivity of the test. RA has a high level of different antibodies, and the report emphasizes the value of analyzing different specificities for the diagnosis. Bitoun et al. immunized Macaque monkeys with citrullinated peptides and showed that the T cell response, but not the B cell response, was mainly directed to citrulline; this is similar to what has been observed in humans. However, in contrast to humans, monkeys with the MHC class II alleles (known to be associated with RA in humans) did not have predisposed T cell or B cell responses to citrullinated peptides. This indicates that we still lack an animal model that accurately reflects the autoimmune process leading to an ACPA response, which is known to occur in RA. Tong et al. highlights another autoantibody in RA, that are likely to also be pathogenic. The target antigen is type II collagen and in the report Tong defines and epitope targeted by such antibodies that is shared between type II and type XI collagen and they also show that both the native and the citrullinated form of the epitope is targeted by antibodies in RA.

## Autoimmune Neurological Diseases

Several articles also focused on autoimmune neurological diseases, mostly on improved diagnostics. Autoantibodies have been shown to be the cause of several neurological diseases, such as anti-NMDA receptor encephalitis (13) or myasthenia gravis. Within this article collection, the role of autoantibodies in “classical” neurodegenerative diseases, such as Parkinson's Disease, is discussed (Jiang et al.). This finding contributes to the current observations that autoantibodies to specific neuronal surface antigens are detected in a number of neuropsychiatric disorders (14). Functional validation of these autoantibodies would change the landscape of treatment for a number of neuropsychiatric diseases.

## Insights Into Pathogenesis

Animal model systems, even with their limitations (15), can significantly contribute to the understanding of disease pathogenesis. Within this Research Topic, two new animal models are described: Zheng et al. describe an immunization-based mouse model for primary Sjögren's Syndrome (Yin et al.). Tong et al. describe the shared epitopes among type XI and type II collagens in mice and humans with arthritis. Furthermore, an immunization-based arthritis model in the macaque (Bitoun et al.) and a model of feline limbic encephalitis (Tröscher et al.) are described within this Research Topic.

Large-scale genetic analyses, such as genome-wide association studies, have provided detailed insights into the underlying genetic association of autoimmune diseases, with the HLA locus as a major risk allele (16–19). Work summarized within this Research Topic demonstrates a co-occurrence of autoimmune diseases, namely, pemphigus and thyroid autoimmunity. Interestingly, the increased prevalence of anti-TPO autoantibodies was associated with the absence of certain HLA alleles and with the presence of non-desmoglein antibodies (Seiffert-Sinha et al.). Overlap at the mRNA expression level is also prevalent in different autoimmune diseases, specifically between pemphigus and systemic lupus erythematosus (SLE) (Sezin et al.). These comparative approaches may be useful to identify novel therapeutic targets that are either specific to one particular autoimmune disease or that may even be effective in the treatment of a specific cluster of autoimmune diseases. Examples of newly identified and validated risk alleles for SLE are described within this Research Topic: Gene expression in the B cells of quiescent SLE patients demonstrated an increased expression of *TRIB1*. To resolve the functional relevance of this gene for SLE pathogenesis, transgenic mice with the B cell-specific overexpression of *Trib1* were generated in the C57BL/6 genetic background. *Trib1* overexpression in B cells led to lower IgG1 concentrations under normal conditions. The immunization of mice with a T cell-dependent antigen also led to lower antigen-specific IgG titers, and the basal or forced anti-dsDNA IgM titers were lower in mice overexpressing *Trib1*. Collectively, these data point toward the *Trib1* regulatory role in autoantibody production in health and in disease (Simoni et al.). Based on the recent discovery of the rare null alleles of deoxyribonuclease 1-like 3 (*DNASE1L3*) and Fc gamma receptor IIB (*FCGR2B*) in SLE patients and genetic mouse models, Weisenburger et al. investigated the functional impact on these 2 genes in mice. For this purpose, mice deficient in both *Dnase1l3*- and *FcgR2b* were generated in the C57BL/6 genetic background. In these mice, high levels of anti-DNA IgG were observed as early as 10 weeks of age. Autoantibody titers in these mice exceeded those observed in 9-month-old NZB/W mice. In conclusion, both genes synergize to promote the IgG anti-DNA autoantibody production by B cells (Weisenburger et al.). For the organ-specific autoimmune disease pemphigus, novel associations with complement genes (Bumiller-Bini et al.) and within the neonatal Fc receptor are described within the Research Topic (Recke et al.).

However, genetics only partially explains disease susceptibility, and (at least in mice) the genetically determined disease susceptibility can be overcome by changing daily habits (20). Indeed, autoantibody production is modulated by environmental factors, such as the diet and the microbiota (Edwards et al.; Petta et al.). Furthermore, gender may have a greater impact on autoantibody production than previously appreciated (Edwards et al.). Within this Research Topic, several manuscripts addressed the contribution of environmental factors on the generation of autoantibodies and/or autoimmune diseases: In their study, Issac et al. showed that the mice who are unable to clear a *Salmonella* infection spontaneously develop anti-dsDNA autoantibodies. This was associated with an increased CD25 expression for both CD4+ and CD8+ T cells. This effect was specific to *Salmonella* infections, as infections caused by other bacteria did not induce autoantibody production (Issac et al.). Two articles demonstrate that pemphigoid can be induced by treatment with gliptins or by physical triggers, such as burns (Gaudin et al.; Mai et al.).

Once autoantibodies are bound to their target antigen, they may induce disease through a variety of mechanisms (21). These are either direct (Fab-mediated effects), such as the induction of aberrant signaling, or alternatively, Fc-mediated events, such as the activation of complement and the engagement of activating Fc-receptors, that drive tissue damage.

In pemphigus, autoantibodies to the desmosomal proteins desmoglein (Dsg) 3 and (often) Dsg 1 cause intraepidermal blistering in the skin and mucous membranes (22). In addition to Dsg 1/3, a wide range of autoantibodies has been identified in pemphigus patients (Amber et al.). The pathogenic relevance of these autoantibodies is not as firmly established as it is for anti-Dsg1/3. However, the injection of IgG from a patient with Dsg 3-reactivity, but not Dsg 1-reactivity, into Dsg 3-deficient mice led to the induction of intraepidermal blistering (23). Recently, a model has been proposed to explain how Dsg- and non-Dsg autoantibodies can lead to intraepidermal blistering. In brief, this model proposes that, depending on the pathogenic activity of all autoantibodies toward the structures on keratinocytes, they are either capable of inducing disease alone or in combination with other autoantibodies (24). The precise mechanisms by which autoantibodies in pemphigus lead to desmosome dysfunction remain to be fully elucidated (Spindler and Waschke): Steric hindrance, i.e., the blockade of homophilic Dsg interactions within the desmosome through autoantibody binding, is believed to be one cause of blistering in pemphigus. Furthermore, Dsg3 is internalized after binding to anti-Dg3 autoantibodies (Schlögl et al.), a process that requires p38 MAPK activation (Cipolla et al.; Vielmuth et al.). In addition to Dsg3 internalization, keratin retraction, induced by pemphigus autoantibodies, has recently been demonstrated to be important in mediating autoantibody-induced cell dissociation (Schlögl et al.).

## Novel Diagnostics / Biomarkers

Precise molecular diagnostics, paired with predictive biomarkers, form the basis of diagnosis as well as the selection of the appropriate treatment for each individual patient. Hence, almost 20% of the articles within the Research Topic “Autoantibodies” have focused on this topic.

The serological detection of autoantibodies is the basis of the diagnosis of many autoimmune diseases (21). Anti-nuclear antibodies (ANAs) are among the most-known autoantibodies. ANAs are associated with several rheumatic diseases, such as systemic lupus erythematosus and systemic sclerosis. However, low titers of ANAs are also present in healthy individuals (25). The gold standard for their detection is by indirect immunofluorescence and incubating the patient serum with Hep-2 cells (26). However, conventional ANA testing requires time, is laborious, and requires microscopy expertise. To overcome these limitations and to standardize and automate ANA indirect IF testing, a fully automated system, which includes staining pattern recognition, was recently developed (27). The workflow and performance characteristics of the fully automated ANA IIF system were compared to manual ANA testing by Ricchiuti et al. The use of fully automated ANA determination has significant labor savings and good concordance with manual ANA readings.

As mentioned above, ANAs are also found in quite a proportion of healthy individuals, ranging from 5 to 30% depending on the population and method used (25, 28–30). This by far exceeds the prevalence of ANA-associated rheumatic diseases. If the target antigen is identified, autoantibodies against DFS70 are often found Interestingly, isolated anti-DFS70 reactivity, which was observed in over 500 serum samples, was not associated with rheumatic disease. Hence, if the dense, fine speckled nuclear pattern, which corresponds to anti-DFS70 reactivity, is observed in Hep cells in ANA testing and anti-DFS70 reactivity is confirmed, then the presence of rheumatic disease is very unlikely (Carter et al.). In contrast, the detection of anti-cN-1A autoantibodies, which are found in 12% of patients with primary Sjögren's syndrome and in 10% of SLE patients, is associated with the presence of other autoimmune diseases (Rietveld et al.).

If an autoimmune disease is suspected but no autoantibodies can be detected by routine methods, these may be identified by applying novel techniques such as the determination of the specific isotypes of the autoantibodies in a suspected case of rheumatoid arthritis (Sieghart et al.) or by the use of a keratinocyte binding assay in a suspected case of pemphigus (Giurdanella et al.). In addition, autoantibodies are also found in certain diseases that are just beginning to be understood to be mediated by autoantibodies. These include neurological conditions (Scharf et al.), chronic obstructive pulmonary disease (Wen et al.), and cardiovascular diseases (Basavalingappa et al.; Ernst et al.; Meier and Binstadt). However, with few exceptions, such as anti-NMDA receptor autoantibodies (31), the pathogenic relevance of these autoantibodies needs to be determined.

Bullous pemphigoid (BP) is the most frequent type of pemphigoid disease (32). BP responds well to systemic (whole body) topical steroid treatment (33). After stopping steroid treatment, relapse occurs in 30–40% of patients (34). Hence, biomarkers that allow for the prediction of relapse would allow for patient selection for whom steroid treatment can be stopped or for determining which patients require prolonged steroid and/or adjuvant treatment. In a retrospective analysis of BP patients, Dr. Koga and colleagues demonstrated that high BP180 autoantibody levels were associated with future relapse. In contrast, age, BP230 antibodies or total IgE levels had no predictive value (35). Researchers from France described elevated anti-type VII collagen autoantibodies, which are the cause of epidermolysis bullosa acquisita (36), in almost half of the BP patients at the time of relapse (Giusti et al.). This is also a retrospective chart analysis with a limited number of patients. However, both studies imply the possibility that predictive biomarkers for BP relapse can be identified. The steps toward this are a joint analysis of retrospective patient cohorts from several departments as well as a prospective diagnostic study.

## Novel Treatments

Based on the understanding of disease pathogenesis, novel treatment targets or therapeutic approaches for autoantibody-mediated diseases have emerged. Within the Research Topic “Autoantibodies,” several articles have focused on new treatments.

The anti-CD20 antibody rituximab has dramatically improved the treatment of several autoantibody-mediated diseases, which was most recently demonstrated in a phase III clinical trial in pemphigus patients (37). Notably, the response to rituximab is not uniform across all autoantibody-mediated diseases, as was demonstrated by the lower efficacy of anti-CD20 treatment in pemphigoid patients when compared to that in pemphigus patients (Lamberts et al.). Rituximab and other emerging treatments to modulate B and plasma cells were the topic of three reviews within the Research Topic (Hofmann et al.; Malkiel et al.; Musette and, Bouaziz). In this Research Topic, Roders et al. also identified SYK as a regulator of B cell activation. Thus, targeting SYK not only affects the effector functions (see below) but also possibly affects the generation of autoantibodies. A different approach to modulate autoantibody concentrations may be to enhance their turnover by inhibiting the neonatal Fc receptor (38) or by selective immunoadsorption using recombinant antigens to specifically elute autoantibodies (Hofrichter et al.).

A blockade of autoantibody functions, either by targeting the Fab or Fc fragments, is another highly interesting treatment approach for autoantibody-mediated diseases. High doses of intravenous immunoglobulins (IVIG) are an effective second- or third-line treatment for a number of autoimmune diseases (39–41). How IVIG mediates the therapeutic effects is controversial (42): One hypothesis claims that all of the therapeutic effects of IVIG are mediated through the inhibition of the neonatal Fc receptor (FcRn) (43). By administering excess IgG, the FcRn becomes saturated, and thus, all IgG molecules (including the autoantibodies) are more rapidly cleared. Others provide compelling evidence that the anti-inflammatory effect of IVIG is mediated by regulating the activation threshold in myeloid effector cells by changing the ratio of activating versus inhibitory FcγR expression (44). This effect required both terminal sialic acid residues at the Fc portion of IgG, as well as the expression of the inhibitory molecule FcγRIIB (45). Finally, the presence of anti-idiotypic antibodies has been reported, specifically, the presence of anti-anti-Dsg 3 autoantibodies in IVIG preparations (46, 47). In this Research Topic, Kamaguchi et al. isolated anti-idiotypic antibodies against type XVII collagen, the major autoantigen in bullous pemphigoid (Liu et al.), and demonstrated a significant inhibitory activity of these antibodies against the pathogenic effects of BP patients' autoantibodies (Kamaguchi et al.).

In addition to the modulation of the Fab function of autoantibodies, their function can also be manipulated by changing the conserved N-linked Fc-glycan attached to the asparagine at position 297 in the constant region of the Fc heavy chain domains (Dekkers et al.). Indeed, the treatment of mice with endo-β-N-acetylglucosaminidase (EndoS), which hydrolyses the β-1,4-di-N-acetylchitobiose core of the N-linked complex type glycan on asparagine 297 (48), suppressed the induction of experimental arthritis (Nandakumar et al.), which was associated with the inhibition of the formation of large immune complexes and was independent of changes in the complement cascade or in antigen binding. The modulation of the conserved IgG's N-glycosylation site may have implications beyond the mere effector functions as was reported by Bartsch et al.: In their work, they demonstrate that antigen-specific sialylated autoantibodies but not non-specific sialylated IgG antibodies, attenuate disease manifestation in experimental lupus and arthritis. The antigen-specific sialylated autoantibodies modulated the B and T cell functions rather than modulating the effector functions of the autoantibodies.

In several autoimmune diseases, such as arthritis and pemphigoid disease, the activation of complement, the binding of immune cells to their immune complexes, and the subsequent intracellular signaling events are important for pathogenesis (21). The generalized inhibition of complement inhibition, which is achieved by the anti-C5 antibody eculizumab (49), is, however, associated with the risk of potentially life-threatening infections (50). These adverse effects could be reduced by restricting the complement inhibition at the site of complement activation or, even more ideally, at the site of pathologic tissue damage. By coupling a cyclic-RGD peptide to a function blocking C5 antibody, the construct is directed to the sites of the damaged endothelial cells. Thus, C5 inhibition preferentially occurs where endothelial damage is present (Durigutto et al.). In addition to the targeted delivery of C5-inhibitory compounds, selectivity may also be achieved by certain complement pathways, which are upregulated in specific diseases. The dissection of the individual contributions of the complement activation cascades in the pemphigoid disease epidermolysis bullosa acquisita [(51); Mihai et al.] demonstrated the predominant role of alternative complement activation and no contribution from the membrane attack complex. Thus, the selective targeting of C1q had therapeutic effects in an animal model of epidermolysis bullosa acquisita (Mihai et al.).

In addition to complement anaphylatoxins, cytokines recruit leukocytes to the sites of autoantibody-induced pathology. Thus, their inhibition has become a well-established therapeutic principle for several chronic inflammatory diseases (52). So far, however, each of the licensed biologics targets a single cytokine. To enhance the anti-inflammatory activity, Abraham et al. used phage display to identify promiscuous chemokine-binding peptides. These bind to a number of pro-inflammatory chemokines, such as CCL2, CCL5, and CXCL9-11. The use of their selected lead compounds in models of autoimmune diseases ameliorated clinical disease manifestation (Abraham et al.). This approach may be applicable in endemic pemphigus foliaceus, where alterations in cytokine and chemokine serum concentrations have previously been noted (Timóteo et al).

Once bound to the immune complexes, a complex signaling cascade is triggered in the leukocytes, which ultimately leads to their activation and subsequent inflammation and tissue damage (53). By contrasting the mRNA expression between inflamed and healthy skin in experimental pemphigoid disease, several hub-genes that potentially contribute to tissue damage in pemphigoid were identified. The spleen tyrosine kinase was among the identified hub-genes. Both LysM-specific SYK knockout mice and mice treated with an inhibitor of the small molecule SYK were completely protected from the induction of experimental pemphigoid disease by autoantibody transfer [Samavedam et al.; (54)]. Corresponding findings were made in a mouse model of arthritis (Németh et al.). Taken together, these findings suggest that targeting SYK is a potential therapeutic approach for a number of autoantibody-mediated diseases. Furthermore, in the pemphigoid mouse model, the inhibition of PI3Kδ also prevented disease onset. Furthermore, pharmacological PI3Kδ inhibition improved clinical disease manifestation when applied in therapeutic, experimental settings (Koga et al.). In addition to these signaling pathways, others also contribute to the pathogenesis of pemphigoid disease, which has recently been reviewed elsewhere (53).

In pemphigus, in addition to the above-described alterations in cell signaling, several lines of evidence suggest that apoptosis contributes to the loss of keratinocyte adhesion and consequently intraepidermal blistering (55). Initially, the contribution of apoptosis was suggested by an increased expression of molecules involved in this process, i.e., different caspases, Fas, as well as FasL (56). Following this concept, the inhibition of the Fas-FasL interaction by a function blocking anti-FasL antibody can inhibit pemphigus IgG-induced pathology *in vitro*. Furthermore, mice lacking the expression of the secreted, soluble FasL do not develop intraepidermal blistering when injected with IgG antibodies from pemphigus patients (Lotti et al.).

All of the abovementioned treatments focused on targeting IgG-mediated autoimmunity. In addition to IgG-mediated autoimmunity, IgA autoantibodies have also long been recognized as pathogenic. However, only recently has attention been attributed to IgE-mediated autoimmunity (Maurer et al.). Despite their presence at high frequencies in many autoimmune diseases, the pathogenic relevance of IgE autoantibodies has thus far not been the focus of studies. In atopic dermatitis, a common, chronic inflammatory skin disease (57), the removal of immunoglobulins by immunoadsorption (and even the selective removal of IgE) has therapeutic effects [(58); Kasperkiewicz et al; Kasperkiewicz et al.], which are comparable to that of biological atopic dermatitis treatment (59). However, immunoadsorption is a very specialized procedure. In addition, especially in the *S. aureus*-colonized skin of atopic dermatitis patients, central intravenous lines may lead to severe infectious complications.

## Concluding Remarks

“Autoantibodies” are a hot research topic, as was reflected by the articles of this Research Topic. Based on an enhanced understanding of disease pathogenesis, as well as the advancement of technology, we are at the verge of developing specific treatments for autoantibody-mediated diseases that may even be able to lead to a cure. This development is, for example, reflected by the use of chimeric autoantigen receptor T cells in the treatment of experimental pemphigus (60). In addition, novel developments in diagnostics now allow for the better diagnosis of patients as well as for the better understanding of the autoimmune nature of diseases, which had not been thought to be caused by an underlying autoimmune mechanism (Scharf et al.). Therefore, even if causal treatment is not possible, we may still be able to carefully select treatments that are tailored to each patient's needs based on the detected biomarkers.

## Author Contributions

All authors listed have made a substantial, direct and intellectual contribution to the work, and approved it for publication.

### Conflict of Interest Statement

RH has received honoraria and/or research grants from Lipum and Astrazeneca and is a founder of the small virtual company Vacara. RL has received honoraria and/or research grants from the following companies: Admirx, Almirall, Amryth, ArgenX, Biotest, Biogen, Euroimmun, Incyte, Immungenetics, Lilly, Novartis, UCB Pharma, Topadur, True North Therapeutics and Tx Cell. The remaining author declares that the research was conducted in the absence of any commercial or financial relationships that could be construed as a potential conflict of interest.

## References

[1] Schmidt E Zillikens D. Pemphigoid diseases. Lancet. (2013) 381:320–32. 10.1016/S0140-6736(12)61140-4 23237497

[2] Hammers CM Stanley JR. Mechanisms of disease: pemphigus and bullous pemphigoid. Annu Rev Pathol. (2016) 11:175–97. 10.1146/annurev-pathol-012615-044313 26907530 PMC5560122

[3] Witte M Zillikens D Schmidt E. Diagnosis of autoimmune blistering diseases. Front. Med. (2018) 5:296. 10.3389/fmed.2018.00296 30450358 PMC6224342

[4] Lamberts A Yale M Grando SA Horváth B Zillikens D Jonkman MF. Unmet needs in pemphigoid diseases: an international survey amongst patients, clinicians and researchers. Acta Derm Venereol. (2018) 99:224–5. 10.2340/00015555-3052 30265372

[5] Alexander T Sarfert R Klotsche J Kühl AA Rubbert-Roth A Lorenz HM. The proteasome inhibitior bortezomib depletes plasma cells and ameliorates clinical manifestations of refractory systemic lupus erythematosus. Ann Rheum Dis. (2015) 74:1474–8. 10.1136/annrheumdis-2014-206016 25710470 PMC4484251

[6] Tipton CM Fucile CF Darce J Chida A Ichikawa T Gregoretti I. Diversity, cellular origin and autoreactivity of antibody-secreting cell population expansions in acute systemic lupus erythematosus. Nat Immunol. (2015) 16:755–65. 10.1038/ni.3175 26006014 PMC4512288

[7] Bolland S Yim YS Tus K Wakeland EK Ravetch JV. Genetic modifiers of systemic lupus erythematosus in FcgammaRIIB(-/-) mice. J Exp Med. (2002) 195:1167–74. 10.1084/jem.20020165 11994421 PMC2193704

[8] Willcocks LC Carr EJ Niederer HA Rayner TF Williams TN Yang W. A defunctioning polymorphism in FCGR2B is associated with protection against malaria but susceptibility to systemic lupus erythematosus. Proc Natl Acad Sci USA. (2010) 107:7881–5. 10.1073/pnas.0915133107 20385827 PMC2867866

[9] Waisberg M Tarasenko T Vickers BK Scott BL Willcocks LC Molina-Cruz A. Genetic susceptibility to systemic lupus erythematosus protects against cerebral malaria in mice. Proc Natl Acad Sci USA. (2011) 108:1122–7. 10.1073/pnas.1017996108 21187399 PMC3024697

[10] Yajima K Nakamura A Sugahara A Takai T. FcgammaRIIB deficiency with Fas mutation is sufficient for the development of systemic autoimmune disease. Eur J Immunol. (2003) 33:1020–9. 10.1002/eji.200323794 12672068

[11] Pisitkun P Deane JA Difilippantonio MJ Tarasenko T Satterthwaite AB Bolland S. Autoreactive B cell responses to RNA-related antigens due to TLR7 gene duplication. Science. (2006) 312:1669–72. 10.1126/science.1124978 16709748

[12] Herrmann M Voll RE Zoller OM Hagenhofer M Ponner BB Kalden JR. Impaired phagocytosis of apoptotic cell material by monocyte-derived macrophages from patients with systemic lupus erythematosus. Arthritis Rheum. (1998) 41:1241–50. 10.1002/1529-0131(199807)41:7<1241::AID-ART15>3.0.CO;2-H 9663482

[13] Venkatesan A Adatia K. Anti-NMDA-receptor encephalitis: from bench to clinic. ACS Chem Neurosci. (2017) 8:2586–95. 10.1021/acschemneuro.7b00319 29077387

[14] Zong S Hoffmann C Mané-Damas M Molenaar P Losen M Martinez-Martinez P. Neuronal surface autoantibodies in neuropsychiatric disorders: are there implications for depression. Front Immunol. (2017) 8:752. 10.3389/fimmu.2017.00752 28725222 PMC5497139

[15] Sundberg JP Schofield PN. Living inside the box: environmental effects on mouse models of human disease. Dis Model Mech. (2018) 11:dmm035360. 10.1242/dmm.035360 30194139 PMC6215423

[16] Vodo D Sarig O Sprecher E. The genetics of pemphigus vulgaris. Front Med. (2018) 5:226. 10.3389/fmed.2018.00226 30155467 PMC6102399

[17] Sajda T Hazelton J Patel M Seiffert-Sinha K Steinman L Robinson W. Multiplexed autoantigen microarrays identify HLA as a key driver of anti-desmoglein and -non-desmoglein reactivities in pemphigus. Proc Natl Acad Sci USA. (2016) 113:1859–64. 10.1073/pnas.1525448113 26831096 PMC4763733

[18] Kim K Bang SY Lee HS Bae SC. Update on the genetic architecture of rheumatoid arthritis. Nat Rev Rheumatol. (2017) 13:13–24. 10.1038/nrrheum.2016.176 27811914

[19] Ghodke-Puranik Y Niewold TB. Immunogenetics of systemic lupus erythematosus: A comprehensive review. J Autoimmun. (2015) 64:125–36. 10.1016/j.jaut.2015.08.004 26324017 PMC4628859

[20] Aqel SI Hampton JM Bruss M Jones KT Valiente GR Wu LC. Daily moderate exercise is beneficial and social stress is detrimental to disease pathology in murine lupus nephritis. Front Physiol. (2017) 8:236. 10.3389/fphys.2017.00236 28491039 PMC5405126

[21] Ludwig RJ Vanhoorelbeke K Leypoldt F Kaya Z Bieber K McLachlan SM. Mechanisms of autoantibody-induced pathology. Front Immunol. (2017) 8:603. 10.3389/fimmu.2017.00603 28620373 PMC5449453

[22] Kasperkiewicz M Ellebrecht CT Takahashi H Yamagami J Zillikens D Payne AS. Pemphigus. Nat Rev Dis Primers. (2017) 3:17026. 10.1038/nrdp.2017.26 28492232 PMC5901732

[23] Vu TN Lee TX Ndoye A Shultz LD Pittelkow MR Dahl MV. The pathophysiological significance of nondesmoglein targets of pemphigus autoimmunity. Development of antibodies against keratinocyte cholinergic receptors in patients with pemphigus vulgaris and pemphigus foliaceus. Arch Dermatol. (1998) 134:971–80. 9722727 10.1001/archderm.134.8.971

[24] Sinha AA Sajda T The evolving story of autoantibodies in Pemphigus vulgaris: development of the “super compensation hypothesis”. Front Med. (2018) 5:218 10.3389/fmed.2018.00218 PMC6102394 30155465

[25] Prussmann J Prussmann W Recke A Rentzsch K Juhl D Henschler R. Co-occurrence of autoantibodies in healthy blood donors.[letter]. Exp Dermatol. (2014) 23:519–21. 10.1111/exd.12445 24816528

[26] Meroni PL Schur PH. ANA screening: an old test with new recommendations. Ann Rheum Dis. (2010) 69:1420–2. 10.1136/ard.2009.127100 20511607

[27] Voigt J Krause C Rohwäder E Saschenbrecker S Hahn M Danckwardt M. Automated indirect immunofluorescence evaluation of antinuclear autoantibodies on HEp-2 cells. Clin Dev Immunol. (2012) 2012:651058. 10.1155/2012/651058 23251220 PMC3502836

[28] Tan EM Feltkamp TE Smolen JS Butcher B Dawkins R Fritzler MJ. Range of antinuclear antibodies in “healthy” individuals. Arthritis Rheum. (1997) 40:1601–11. 10.1002/1529-0131(199709)40:9<1601::AID-ART9>3.0.CO;2-T 9324014

[29] Satoh M Chan EK Ho LA Rose KM Parks CG Cohn RD. Prevalence and sociodemographic correlates of antinuclear antibodies in the United States. Arthritis Rheum. (2012) 64:2319–27. 10.1002/art.34380 22237992 PMC3330150

[30] Semchuk KM Rosenberg AM McDuffie HH Cessna AJ Pahwa P Irvine DG. Antinuclear antibodies and bromoxynil exposure in a rural sample. J Toxicol Environ Health A. (2007) 70:638–57. 10.1080/15287390600974593 17365618

[31] Planagumà J Leypoldt F Mannara F Gutiérrez-Cuesta J Martín-García E Aguilar E. Human N-methyl D-aspartate receptor antibodies alter memory and behaviour in mice. Brain. (2015) 138:94–109. 10.1093/brain/awu310 25392198 PMC4285189

[32] Hübner F Recke A Zillikens D Linder R Schmidt E. Prevalence and age distribution of pemphigus and pemphigoid diseases in Germany. J Invest Dermatol. (2016) 136:2495–8. 10.1016/j.jid.2016.07.013 27456755

[33] Joly P Roujeau JC Benichou J Picard C Dreno B Delaporte E. A comparison of oral and topical corticosteroids in patients with bullous pemphigoid. N Engl J Med. (2002) 346:321–7. 10.1056/NEJMoa011592 11821508

[34] Joly P Roujeau JC Benichou J Delaporte E D'Incan M Dreno B. A comparison of two regimens of topical corticosteroids in the treatment of patients with bullous pemphigoid: a multicenter randomized study. J Invest Dermatol. (2009) 129:1681–7. 10.1038/jid.2008.412 19177141

[35] Koga H Teye K Ishii N Ohata C Nakama T. High index values of enzyme-linked immunosorbent assay for BP180 at baseline predict relapse in patients with bullous pemphigoid. Front Med. (2018) 5:139. 10.3389/fmed.2018.00139 29868591 PMC5954083

[36] Koga H Prost Squarcioni C Iwata H Jonkman MF Ludwig RJ Bieber K. Epidermolysis bullosa acquisita: The 2019 update. Front Med. (2018) 5:362. 10.3389/fmed.2018.00362 30687710 PMC6335340

[37] Joly P Maho-Vaillant M Prost-Squarcioni C Hebert V Houivet E Calbo S. First-line rituximab combined with short-term prednisone versus prednisone alone for the treatment of pemphigus (Ritux 3): a prospective, multicentre, parallel-group, open-label randomised trial. Lancet. (2017) 389:2031–40. 10.1016/S0140-6736(17)30070-3 28342637

[38] Lee J Werth VP Hall RP Eming R Fairley JA Fajgenbaum DC. Perspective from the 5th International Pemphigus and Pemphigoid Foundation scientific conference. Front Med. (2018) 5:306. 10.3389/fmed.2018.00306 30467542 PMC6236000

[39] Iwata H Vorobyev A Koga H Recke A Zillikens D Prost-Squarcioni C. Meta-analysis of the clinical and immunopathological characteristics and treatment outcomes in epidermolysis bullosa acquisita patients. Orphanet J Rare Dis. (2018) 13:153. 10.1186/s13023-018-0896-1 30180870 PMC6122731

[40] Ishii N Hashimoto T Zillikens D Ludwig RJ. High-dose intravenous immunoglobulin (IVIG) therapy in autoimmune skin blistering diseases. Clin Rev Allergy Immunol. (2010) 38:186–195. 10.1007/s12016-009-8153-y 19557317

[41] Amagai M Ikeda S Shimizu H Iizuka H Hanada K Aiba S. A randomized double-blind trial of intravenous immunoglobulin for pemphigus. J Am Acad Dermatol. (2009) 60:595–603. 10.1016/j.jaad.2008.09.052 19293008

[42] Schwab I Nimmerjahn F. Intravenous immunoglobulin therapy: how does IgG modulate the immune system. Nat Rev Immunol. (2013) 13:176–89. 10.1038/nri3401 23411799

[43] Li N Zhao M Hilario-Vargas J Prisayanh P Warren S Diaz LA. Complete FcRn dependence for intravenous Ig therapy in autoimmune skin blistering diseases. J Clin Invest. (2005) 115:3440–50. 10.1172/JCI24394 16284651 PMC1280965

[44] Kaneko Y Nimmerjahn F Ravetch JV. Anti-inflammatory activity of immunoglobulin G resulting from Fc sialylation. Science. (2006) 313:670–3. 10.1126/science.1129594 16888140

[45] Schwab I Mihai S Seeling M Kasperkiewicz M Ludwig RJ Nimmerjahn F. Broad requirement for terminal sialic acid residues and FcgammaRIIB for the preventive and therapeutic activity of intravenous immunoglobulins *in vivo*. Eur J Immunol. (2014) 44:1444–53. 10.1002/eji.201344230 24505033

[46] Mimouni D Blank M Payne AS Anhalt GJ Avivi C Barshack I. Efficacy of intravenous immunoglobulin (IVIG) affinity-purified anti-desmoglein anti-idiotypic antibodies in the treatment of an experimental model of pemphigus vulgaris. Clin Exp Immunol. (2010) 162:543–9. 10.1111/j.1365-2249.2010.04265.x 20964642 PMC3026558

[47] Mimouni D Blank M Ashkenazi L Milner Y Frusic-Zlotkin M Anhalt GJ. Protective effect of intravenous immunoglobulin (IVIG) in an experimental model of pemphigus vulgaris. Clin Exp Immunol. (2005) 142:426–32. 10.1111/j.1365-2249.2005.02947.x 16297153 PMC1809530

[48] Collin M Olsen A. EndoS, a novel secreted protein from Streptococcus pyogenes with endoglycosidase activity on human IgG. EMBO J. (2001) 20:3046–55. 10.1093/emboj/20.12.3046 11406581 PMC150189

[49] Zuber J Fakhouri F Roumenina LT Loirat C Frémeaux-Bacchi V French SGFAHUS. Use of eculizumab for atypical haemolytic uraemic syndrome and C3 glomerulopathies. Nat Rev Nephrol. (2012) 8:643–57. 10.1038/nrneph.2012.214 23026949

[50] Benamu E Montoya JG. Infections associated with the use of eculizumab: recommendations for prevention and prophylaxis. Curr Opin Infect Dis. (2016) 29:319–29. 10.1097/QCO.0000000000000279 27257797

[51] Mihai S Chiriac MT Takahashi K Thurman JM Holers MV Zillikens D. The alternative pathway in complement activation is critical for blister induction in experimental epidermolysis bullosa acquisita. J Immunol. (2007) 178:6514–21. 10.4049/jimmunol.178.10.6514 17475881

[52] Reichert JM. Marketed therapeutic antibodies compendium. MAbs. (2012) 4:413–5. 10.4161/mabs.19931 22531442 PMC3355480

[53] Ludwig RJ Signaling and targeted-therapy of inflammatory cells in epidermolysis bullosa acquisita. Exp Dermatol. (2017). 26:1179–86. 10.1111/exd.13335 28266741

[54] Németh T Virtic O Sitaru C Mócsai A. The Syk tyrosine kinase is required for skin inflammation in an *in vivo* mouse model of epidermolysis bullosa acquisita. J Invest Dermatol. (2017) 137:2131–9. 10.1016/j.jid.2017.05.017 28576735 PMC5624865

[55] Schmidt E Waschke J. Apoptosis in pemphigus. Autoimmun Rev. (2009) 8:533–7. 10.1016/j.autrev.2009.01.011 19189866

[56] Pacheco-Tovar MG Avalos-Díaz E Vega-Memije E Bollain-y-Goytia JJ López-Robles E Hojyo-Tomoka MT. The final destiny of acantholytic cells in pemphigus is Fas mediated. J Eur Acad Dermatol Venereol. (2009) 23:697–701. 10.1111/j.1468-3083.2009.03162.x 19470049

[57] Leung DY Bieber T. Atopic dermatitis. Lancet. (2003) 361:151–160. 10.1016/S0140-6736(03)12193-9 12531593

[58] Wegner J Weinmann-Menke J von Stebut E. Immunoadsorption for treatment of severe atopic dermatitis. Atheroscler Suppl. (2017) 30:264–70. 10.1016/j.atherosclerosissup.2017.05.043 29096848

[59] Beck LA Thaci D Hamilton JD Graham NM Bieber T Rocklin R. Dupilumab treatment in adults with moderate-to-severe atopic dermatitis. N Engl J Med. (2014) 371:130–9. 10.1056/NEJMoa1314768 25006719

[60] Ellebrecht CT Bhoj VG Nace A Choi EJ Mao X Cho MJ. Reengineering chimeric antigen receptor T cells for targeted therapy of autoimmune disease. Science. (2016) 353:179–184. 10.1126/science.aaf6756 27365313 PMC5343513

